# Electroretinograms recorded with skin electrodes in silicone oil-filled eyes

**DOI:** 10.1371/journal.pone.0216823

**Published:** 2019-05-31

**Authors:** Kimitake Ozaki, Yuji Yoshikawa, Sho Ishikawa, Takeshi Katsumoto, Masayuki Shibuya, Takuhei Shoji, Hiromi Kondo, Soiti Matsumoto, Kei Shinoda

**Affiliations:** 1 Department of Ophthalmology, Saitama Medical University, Iruma-gun, Saitama, Japan; 2 Matsumoto Eye Clinic, Awa City, Tokushima, Japan; University of Houston, College of Optometry, UNITED STATES

## Abstract

**Purpose:**

To assess the physiology of the retina by electroretinography (ERG) with skin electrodes in eyes that had undergone vitrectomy with silicone oil (SO) tamponade.

**Design:**

Retrospective case series.

**Method:**

ERGs were recorded from eleven eyes with complex vitreoretinal disorders and from the normal fellow eyes. The affected eyes underwent pars plana vitrectomy (PPV) with SO tamponade. ERGs were recorded before and after the SO was removed. The amplitudes and implicit times of the a- and b-waves of the affected eyes were compared to those of the normal fellow eyes. In addition, the ratios of the amplitudes of the b-waves of the affected eyes to those of the fellow eyes were compared before and after the SO was removed.

**Results:**

ERGs were recordable from 7 eyes (63.6%) before the SO was removed and 11 eyes (100%) after the SO was removed. The a- and b-wave amplitudes were significantly smaller in the affected eyes than those of the fellow eyes at the baseline. The b-wave amplitude before the removal of the SO was significantly and positively correlated with that after the SO removal. The ratios of the b-waves of the affected/normal fellow eye significantly increased after the SO was removed.

**Conclusion:**

The results indicate that ERGs picked up by skin electrode can be used to assess the physiology of the retina in eyes with a SO tamponade. The amplitude of the b-waves of the ERGs in silicone-filled eyes can be used to predict the amplitude after the silicone is removed.

## Introduction

Silicone oil (SO) was first used as a retinal tamponade in cases of complex retinal detachments (RDs) by Cibis et al [1–3]. It was also used as a tamponade in complicated vitreoretinal disorders such as proliferative vitreoretinopathy (PVR) [2,3], proliferative diabetic retinopathy (PDR) [2,3], giant retinal tears [3,4], traumatic injuries [3,5], and viral retinitis [3,6]. Its optical clarity allowed clinicians to examine the retina postoperative by ophthalmoscopy and optical coherence tomography.

Electroretinographic (ERG) examinations of the retina are performed to determine the physiological status of the retina in eyes with suspected retinal pathology and occasionally after intraocular surgery. However, ERGs are not performed routinely after intraocular surgeries because ERG recordings require the use of contact lens electrodes which can be a risk for corneal abrasions [7] and infections for which fiber or skin electrode can be alternative methods [8,9]. Another disadvantage of electrophysiological examinations after intraocular surgery is that the vitreous cavity may be filled with a non-conductive tamponade such as SO which has been reported to cause a reduction of the ERGs [10–15].

Recordings of ERGs before and after the removal of SO has been done under experimental [16] and clinical [15,17,18] conditions. Meredith et al. [16] performed pars plana vitrectomy (PPV) bilaterally followed by injection of SO into the vitreous cavity of one eye of rabbits. They reported that the a- and b-waves amplitudes of the ERGs of both eyes were reduced during the early postoperative period. With increasing time, there was a recovery of the amplitudes to the baseline values in both eyes. In contrast, an increase in the ERGs has been reported in patients shortly after the SO was removed [14,15,19], and this increase was attributed to the removal of the non-conductive SO.

The RETeval system (LKC Technologies Inc., Gaithersburg, MD; Welch Allyn, Inc., Skaneateles Falls, NY) is a handheld, portable ERG device that uses skin electrodes to pick up the ERGs. The recordings can be done rapidly, and the skin electrodes reduce the risk of corneal abrasion and infections. Thus, it allows clinicians to assess the physiology of the retina shortly after any type of intraocular surgery [20–23].

These properties prompted us to evaluate the retinal function by the RETeval system in eyes filled with SO before and after the silicone oil was removed. The relationships of the ERG findings to the clinical conditions were determined.

## Subjects and methods

All of the participants had undergone PPV with SO tamponade at the Saitama Medical University Hospital in Saitama, Japan from March 2017 to June 2018. All of the patients had signed a written informed consent after the nature and possible complications of the surgery had been explained. This was a retrospective study that was conducted in accordance with the tenets of Declaration of Helsinki and was approved by the Ethics Committee of Saitama Medical University, Saitama, Japan (ID number: 18067.01).

Seventeen eyes of 11 patients with complex vitreoretinal disorders were studied. There were 5 men and 6 women who had undergone pars plana vitrectomy (PPV) with purified SO as a tamponade (SILIKON1000, Alcon Japan Ltd, Tokyo, Japan). The median (25th, 75th percentiles) age of the patients was 58.0 (52.0, 70.0) years. The SO was removed when the retinal reattachment appeared stable with no signs of active vitreoretinal pathology. The medical records were reviewed to determine; the vitreoretinal pathology necessitating the PPV with SO tamponade, best-corrected visual acuity (BCVA) before and after the SO removal, attached or detached retina, and presence of posterior synechia of the iris.

The ERGs were recorded with the RETeval system, and the recording conditions conformed to the standards of the International Society for Clinical Electrophysiology of Vision (ISCEV) [24]. ERGs were recorded before and after the SO was removed in all eyes. The ERGs were recorded after 20 minutes of dark-adaptation, and the combined rod and cone responses were picked-up by a sensor strip skin electrode affixed to the lower eyelid of both eyes. The strips included the active, reference, and ground electrodes. A mini Ganzfeld dome was placed in front of the eye and a 3.0 cd·s/m^2^ flash without background light was delivered to elicit the ERGs. The patients were instructed to fixate a point within the dome, and the fixation was monitored by an infrared camera.

The implicit times and amplitudes of a- and b-waves were automatically analyzed by the software integrated in the RETeval system. The analyses were performed before and after the SO was removed. The ratios of the amplitudes and implicit times of the a- and b-waves of the affected eye to that in the of the fellow eye were calculated. The ratio of the implicit times for eyes with non-recordable ERGs were excluded.

### Statistical analyses

The amplitudes and implicit times of the a- and b-waves of the affected eyes were compared to that of the normal fellow eyes using the Wilcoxon signed rank test. The amplitudes and implicit times before and after the SO were removed were compared using Wilcoxon signed rank test, and the relationships were analyzed using Spearman's Rank Order Correlations. The ratios of the amplitudes and implicit times before and after the SO removal were compared using Wilcoxon signed rank test. Spearman's rank order correlations were performed to investigate the relationship between the amplitudes of the a- and b-waves before and after the SO was removed. The decimal BCVA was converted to the logarithm of minimum angle of resolution (logMAR) for the statistical analyses. Comparisons before and after the SO was removed was done with Wilcoxon signed rank test. Visual acuities of ‘counting fingers’, ‘hand movements’, and ‘light perception’ were assigned values of 2.0, 2.4, and 2.7 logMAR units, respectively [25]. A *P* <0.05 was taken to be statistically significant.

## Results

The demographics and clinical information of the patients are summarized in **Table 1**. The vitreoretinal pathologies leading to the vitrectomy with SO tamponade were RD associated with multiple breaks, inferior breaks, or trauma, and PVR in 5 (45%) of the 11 eyes **(Table 2)**. The retina remained attached after the SO was removed in all 11 eyes. The median (25th, 75th percentiles) duration of the SO tamponade was 119 (97, 140) days. The median (25th, 75th percentiles) interval from the SO removal to the second ERG recording was 62 (25, 111) days.

**Table 1:** Demographics of patients.

age (years)	58.0 (52.0, 70.0)
**sex (F/M)**	**5 / 6**
**log MAR**	
** before SOR**	**0.82 (0.52, 1.00)**
** after SOR**	**0.70 (0.22, 1.22)**
Duration of SO tamponade until first ERG recording (days)*	**119 (97, 140)**
Period between SOR and second ERG recording (days)**	**62 (25, 111)**
**Posterior synechia of the iris (+/-)**	**4/7**
**Vitreoretinal pathology**	
RRD***	**11**

Data is shown as median (25th, 75th percentiles).

F:female, M:male, SOR:silicone oil removal

log MAR:logarythm of minimal angular resolution.

*Duration of SO tamponade until first ERG recording (days) means duration between pre-SOR ERG and SOR (days).

**Period between SOR and second ERG recording (days) means duration between SOR and post-SOR ERG recording (days).

RRD:rhegmatogeneous retinal detachment

***: including 5 eyes with proliferative vitreoretinopathy

**Table 2:** Clinical findings and ERG parameters in individual patients.

No.	bilatelarity	age (years old)	gender	vitreoretinal pathology	indication for SO use	intracameral SO	encircling	number of preSOR surgeries	synechia		Period between SOR and ERG recordings (days)		LogMAR		Ratio of ERG parameter (affected eye/ fellow eye)								Group
															before SOR				after SOR				
															amplitude		implicit time		amplitude		implicit time		
									before SOR	after SOR	before SOR	after SOR	before SOR	after SOR	a wave	b wave	a wave	b wave	a wave	b wave	a wave	b wave	
**1**	**R**	**57**	**F**	**RRD**	**indferior, multiple breaks**	**-**	**+**	**1**	**-**	**-**	**125**	**26**	**1.52**	**1.70**	**0.07**	**0.08**	**1.36**	**0.96**	**0.22**	**0.48**	**1.42**	**1.48**	**Group 1**
**2**	**L**	**52**	**M**	**PVR**	**PVR**	**+**	**+**	**2**	**-**	**-**	**97**	**76**	**0.52**	**0.30**	**0.64**	**0.51**	**1.04**	**1.08**	**0.55**	**0.47**	**1.36**	**0.95**	**Group 1**
**3**	**L**	**72**	**F**	**RRD**	**indferior break**	**-**	**-**	**2**	**-**	**-**	**112**	**111**	**0.05**	**0.22**	**0.87**	**0.69**	**0.99**	**0.99**	**1.15**	**1.07**	**0.95**	**1.18**	**Group 1**
**4**	**R**	**58**	**M**	**RRD**	**indferior, multiple breaks**	**-**	**-**	**1**	**-**	**-**	**84**	**13**	**0.52**	**0.22**	**0.81**	**0.94**	**1.29**	**1.09**	**1.83**	**1.63**	**1.26**	**0.78**	**Group 1**
**5**	**R**	**48**	**F**	**PVR**	**PVR**	**+**	**-**	**2**	**+**	**-**	**99**	**25**	**1.00**	**0.70**	**0.00**	**0.00**	**n.a.**	**n.a.**	**0.96**	**0.97**	**1.52**	**1.11**	**Group 2**
**6**	**R**	**56**	**F**	**RRD**	**difficulty in keeping prone position**	**-**	**+**	**2**	**+**	**-**	**71**	**33**	**0.82**	**1.22**	**0.00**	**0.00**	**n.a.**	**n.a.**	**0.74**	**0.69**	**1.10**	**0.88**	**Group 2**
**7**	**R**	**70**	**M**	**PVR**	**PVR**	**-**	**-**	**1**	**+**	**-**	**122**	**62**	**2.00**	**1.30**	**0.00**	**0.00**	**n.a.**	**n.a.**	**0.70**	**0.54**	**1.47**	**1.17**	**Group 2**
**8**	**R**	**67**	**M**	**PVR**	**PVR**	**-**	**+**	**3**	**-**	**-**	**140**	**111**	**0.82**	**0.82**	**0.00**	**0.00**	**n.a.**	**n.a.**	**0.70**	**0.51**	**1.15**	**0.79**	**Group 2**
**9**	**R**	**52**	**F**	**PVR**	**PVR**	**+**	**-**	**2**	**+**	**-**	**458**	**104**	**1.00**	**0.70**	**0.42**	**0.22**	**1.20**	**0.91**	**0.60**	**0.54**	**1.12**	**0.95**	**Group 1**
**10**	**L**	**63**	**M**	**RRD**	**post ocular trauma**	**-**	**-**	**1**	**-**	**-**	**119**	**287**	**0.40**	**0.22**	**0.48**	**0.52**	**1.26**	**1.00**	**1.16**	**0.87**	**1.06**	**1.20**	**Group 1**
**11**	**L**	**73**	**M**	**RRD**	**post ocular trauma**	**+**	**-**	**1**	**-**	**-**	**198**	**10**	**0.70**	**1.00**	**0.70**	**0.60**	**0.86**	**1.02**	**1.49**	**1.50**	**0.99**	**1.11**	**Group 1**

ERG: electroretinogram, SOR:silicone oil removal, Log MAR:logarythm of minimal angular resolution, R:right, L:left

RRD: rhegmatogeneous retinal detachment, PVR: proliferative vitreoretinopathy, F: female, M: male, n.a.: not applicable because the implicit time before SOR was unmeasurable.

Group 1: eyes which showed recordable ERG (+) both before and after SOR, Group 2: eyes which showed non-recordable ERG before SOR and recordable ERG after SOR.

Four eyes had a posterior synechia which prevented a maximal dilation of the pupils before the SO was removed **(Tables 1 and 2).**

The median BCVA was 0.82 (0.52, 1.00) logMAR units with a range of 0.05 to 2.00 logMAR units before the SO removal, and it was 0.70 (0.22, 1.22) logMAR units with a range of 0.22 to 1.70 logMAR units after the removal of the SO (*P* = 0.461).

Information of the time of ERG recordings and each ERG parameter in individual patients are presented in **Table 2.** ERGs were recordable from 7 of the 11 eyes (63.6%) before the SO was removed and 11 of the 11 eyes (100%) after the SO was removed. No ERG responses were observed in 4 eyes (36.4%) before the SO was removed and are designated as ERG (-).

**Fig 1:**
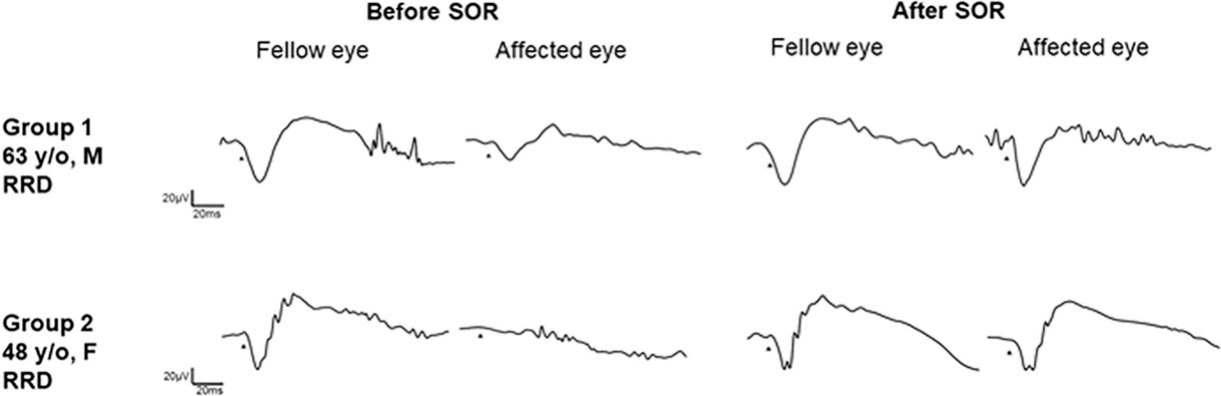
Electroretinograms (ERGs) of representative cases from each group. The combined rod and cone responses before and after silicone oil (SO) removal are shown. In Group 1, this is a case of rhegmatogenous retinal detachment (RRD). A clear waveform was observed in the SO filled eye and the amplitudes of the a- and b-waves increased after the SO removal. In Group 2, this is a case of RRD in which pupillary dilatation was limited due to a posterior synechia of the iris. No response was observed before the SO removal and a waveform was recorded after SO removal and the posterior synechia was released. SOR, silicone oil removal; RRD, rhegmatogenous retinal detachment; M, male; F, female.

All eyes were classified into two groups according to presence of ERGs or ERGs (+) or ERG (-) before and after the SO removal (**Fig 1, Table 2**). Group 1 was comprised of 7 eyes which were ERG (+) before and after the SO removal and Group 2 was comprised of 4 eyes which were ERG (-) before and ERG (+) after the SO removal. None of the eyes with ERG (+) before the SO removal had ERG (-) after the removal, and none of the eyes had ERG (-) both before and after the SO removal.

**Fig 2:**
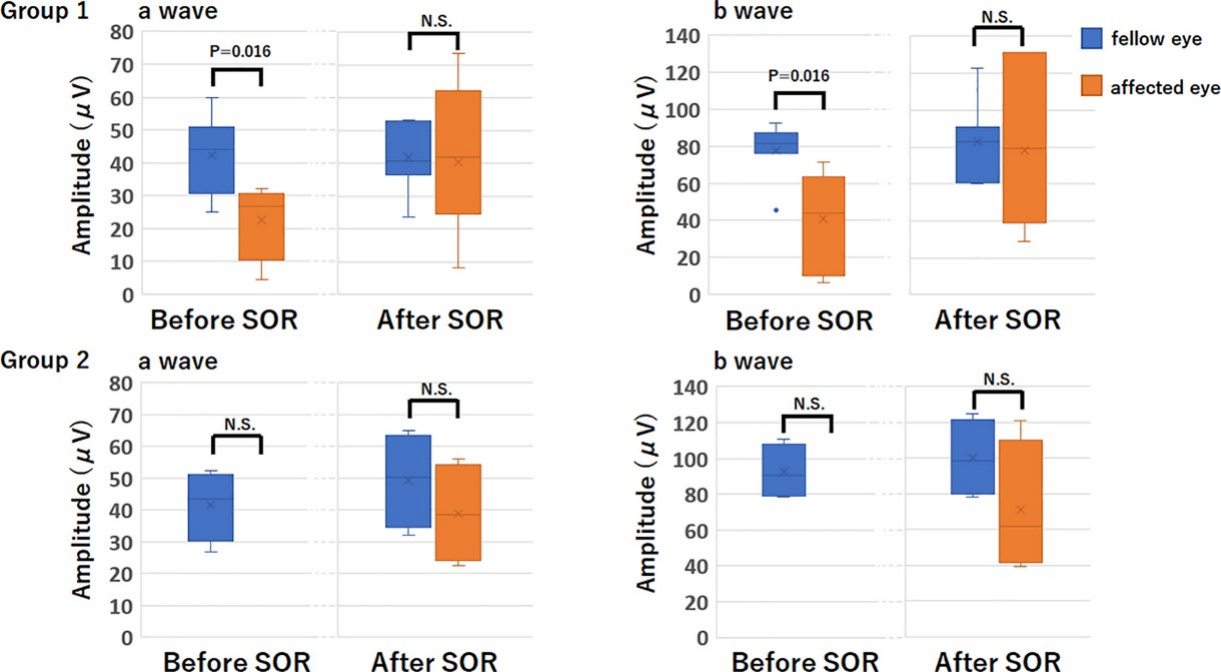
Amplitude of a- and b-waves before and after silicone oil (SO) removal in Groups 1 (upper) and 2 (lower). In Group 1, the amplitudes of the a- and b-waves were significantly reduced before SO removal compared to the fellow eye. However, after SO removal, the amplitudes of the a- and b-waves were not significant different between the two eyes. In Group 2, no significant difference was observed between the amplitudes of the a- and b-waves before and after SO removal. SO, silicone oil; SOR, silicone oil removal; NS, not significant.

In Group 1, the amplitudes of a- and b-waves were significantly smaller than that of the fellow eye before the SO removal (**Fig 2**). However, the amplitudes of the a- and b-waves were not significantly different from that of the fellow eyes after the SO was removed (**Fig 2**). In Groups 2, there was a trend for the difference in the amplitudes of the a- and b-waves between the affected and normal fellow eye before and after the SO removal to be smaller, but it did not reach statistical significance probably due to the small sample number (*P* = 0.125 for each, **Fig 2**). In cases where the ERGs were at noise level, the amplitudes and implicit times were unmeasurable. Thus, the implicit times were not suitable for statistical analysis. We therefore performed statistical analyses only on the amplitudes.

The ratios (affected eye/normal fellow eye) of the amplitudes and implicit times are shown in **Table 3**. In Group 1, the ratios for both the a- and b-waves amplitudes after the SO was removed were significantly increased compared to those before the SO removal. In Group 2, the ratio for both the a- and b-wave amplitudes after the SO removal was increased compared to those before the removal although the anatomic condition and retinal attachment status remained unchanged, but it did not reach statistical significance probably due to small sample number.

**Table 3:** The ratio of affected eye on fellow eye of the electroretinographic parameters.

**amplitude**		** **	** **	** **	** **	** **	** **
** **	** **	**before SOR**		**after SOR**		**comparison before and after SOR (p value**^**a**^**)**	
	**n**	**a wave**	**b wave**	**a wave**	**b wave**	**a wave**	**b wave**
**all**	**11**	**0.42 (0.00, 0.70)**	**0.22 (0.00, 0.60)**	**0.74 (0.60, 1.16)**	**0.69 (0.51, 1.07)**	***0*.*002***	***0*.*002***
**group I**	**7**	**0.64 (0.42, 0.81)**	**0.52 (0.22, 0.69)**	**1.15 (0.55, 1.49)**	**0.87 (0.48, 1.50)**	***0*.*031***	***0*.*031***
**group II**	**4**	**0.00 (0.00, 0.00)**	**0.00 (0.00, 0.00)**	**0.72 (0.70, 0.90)**	**0.62 (0.52, 0.90)**	***0*.*125***	***0*.*125***
**implicit time**		** **	** **	** **	** **	** **	** **
** **	** **	**before SOR**		**after SOR**		**comparison before and after SOR (p value**^**a**^**)**	
** **	**n**	**a wave**	**b wave**	**a wave**	**b wave**	**a wave**	**b wave**
**all**	**11**	**1.20 (0.99, 1.29)**	**1.00 (0.96, 1.08)**	**0.74 (0.60, 1.16)**	**1.15 (1.06, 1.42)**	**0.940**	**0.469**
**group I**	**7**	**1.20 (0.99, 1.29)**	**0.99 (0.96, 1.08)**	**1.12 (0.99, 1.36)**	**1.11 (0.95, 1.20)**	**0.940**	**0.469**
**group II**	**4**	**n.a.**	**n.a.**	**1.31 (1.11, 1.51)**	**0.99 (0.81, 1.16)**	**n.a.**	**n.a.**

Data are presented as median (25th, 75th percentiles), SOR; silicone oil removal, n.a.; not applicable, a; wilcoxom signed rank test

**Fig 3:**
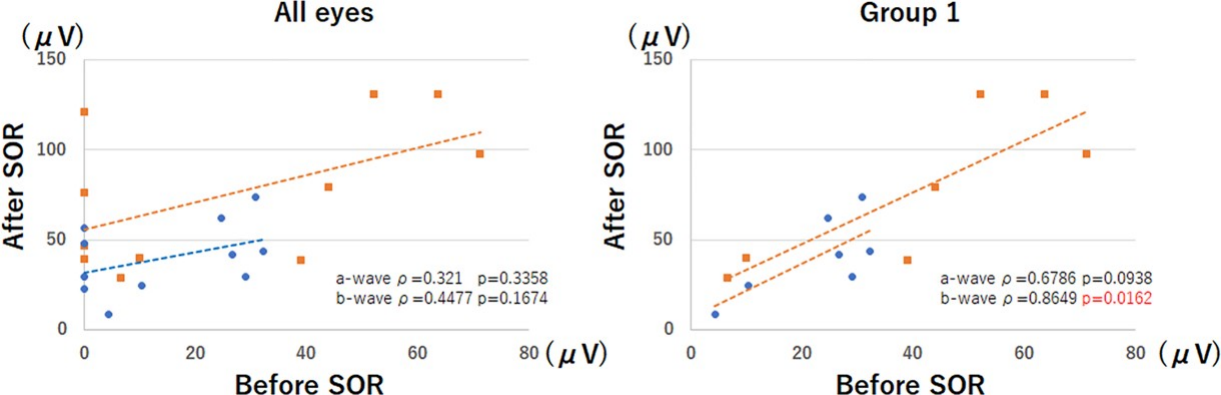
Amplitudes of the a- and b-waves before and after silicone oil (SO) removal. There was a significant positive correlation between the b-wave amplitudes before and after SO removal for the 7 eyes in Group 1 (ρ = 0.8649, *P* = 0.0162). SOR, silicone oil removal.

When an ERG was recordable from the SO-filled eyes, the amplitudes of the b-waves were significantly correlated with the amplitudes after the removal (ρ = 0.8649, *P* = 0.0162, Spearman's Rank Order Correlation; **Fig 3**) in Group 1. There was no significant correlation in the ratios of the a- and b-waves and the duration of the SO tamponade.

A posterior synechia was found in 4 eyes before the SO was removed, and three of them were among the 4 eyes with ERG (-). The percentage of eyes with posterior synechia was higher in eyes with ERG (-) than in eyes with ERG (+) but the difference was not significant, (*P* = 0.09, Fisher’s exact test).

In the 4 eyes with a posterior synechia before the SO was removed, the posterior synechia was released during the SO removal surgery and the ERG responses increased, or small ERGs were present after the SO removal (**Fig 1**).

The frequency of anterior chamber SO migration and existence of encircling (p = 1.00, p = 0.58, respectively, Fisher’s exact test), and the number of surgeries before SO removal (p = 0.34, Wilcoxon signed rank test) were not significantly different between the groups.

## Discussion

The results showed that quantitative ERG evaluations were possible with skin electrodes in about one-half of the SO-filled eyes. In eyes where the ERGs were present before the SO removal, an increase in the amplitudes of the ERGs can be expected after the removal of the SO. All eyes in which the ERGs were extinguished before the SO was removed had ERGs after the SO removal, and the release of the posterior synechia may be one of the reasons for the ERG responses after the SO removal.

The amplitudes of the ERGs were smaller than that of the fellow eye in all eyes before the SO removal but increased after the removal suggesting that the reduced amplitudes in SO filled eyes was due to the non-conductive properties of the SO. In addition, a posterior synechia of the iris which was occasionally present in eyes that had undergone PPV with tamponade [26], may have contributed to the reduced response by reducing the level of retinal illuminance.

The SO was removed on days 71 to 458 after the SO had been inserted. Because the minimum duration for photoreceptor recovery after retinal reattachment was reported to be about four weeks [27,28], the photoreceptors should have largely recovered and therefore the ERG responses before the SO removal can be considered a reliable baseline for comparisons to those after the SO removal.

Frumar et al. [18] recorded ERGs from 10 eyes before and after SO removal. Because the conditions for the ERG recordings, e.g., stimulus parameters, recording conditions, state of adaptations were not stated, and statistical analyses were not performed, direct comparisons between their findings and ours are not possible. However, they reported that ERGs could be recorded from SO-filled eye and the amplitudes changed after the SO was removed. The mean a-wave amplitude before the SO was removed was 40 μV, and it was 27 μV at 3 days and 88 μV one month after the SO removal. The b-wave amplitude before SO was removed was 94 μV, just after SO was removed was 148 μV, and 3 months after SO was removed was 184 uV. They stated that it was possible to record sizable ERG even in the presence of a large body of a non-conductive agent. They explained this by stating that a small but significant conducting path was present between the retinal surface and the cornea which allowed the recording of the ERGs. They assumed that the path is represented by a thin film of fluid between the retina and the tamponading bubble. Our results agree with their findings in that sizable ERGs can be recorded even from SO-filled eyes.

When recording ERGs with RETeval, the skin electrodes are placed inferior to the lower margin of the eye lid. Therefore, the skin electrodes are located near the aqueous humor even in SO-filled eye when patients are sitting during the recordings. In contrast, contact lens electrodes are located near the SO which could further isolate the ERG signals. In addition, we found that the ERGs before and after the SO removal were significantly and positively correlated. This suggests that when ERGs can be recorded before the SO removal, the amplitudes of the ERGs can predict the amplitudes after the SO removal.

It has been reported [29] that a SO tamponade of more than 9 months will cause alterations of the retinal oxygen saturation and a narrowing of the retinal arterioles which may further interfere with the oxygen delivery to the retina. Thus, the influence of the alterations of retinal saturation of the ERG responses in our cases is believed to be minimal which is supported by the absence of significant correlations between the ratios and the durations of the SO tamponade. However, it should also be remembered that the ERG responses can gradually decrease in eyes with long-term SO tamponade.

The RETeval system can be used on eyes without pupillary dilation because the device delivers a stimulus with constant retinal illuminance (photopic Td-s) by adjusting the luminance (photopic cd-s/m^2^) to compensate for changes in the pupillary area (mm^2^). However, it has been reported that the effective retinal illuminance of the stimulus delivered by the RETeval system decreases for large pupil sizes [20]. For eyes with pupil diameter less than approximately 6.5 mm, the RETeval system delivers a stimulus with constant retinal illuminance. In our study, the mydriasis-free mode was not used because the posterior synechia in the affected eyes could have interfered with a full mydriasis.

Our study has several limitations. First, this was a retrospective study with its shortcomings. Second, the pupil size when ERG was recorded was not available. Because our results suggest considerable influence of the pupil size on the ERGs recorded from SO filled eye, further studies with accurate pupil size and/or mydriasis-free mode recordings are needed. Third, the sample size was small, and analysis on eyes with RRD and PVR could not be done separately. Because the underlying disease may affect the ERG responses, homogeneous vitreoretinal diseases, such as retinal detachments with minimal proliferative factor might be better. On the other hand, our study reflects the daily clinical situation and would be applicable to clinical practice. Fourth, the fellow eyes had not undergone surgery so one may argue that it is not completely comparable, and perhaps eyes undergoing vitrectomy without SO would be a better control group. However, it is difficult to collect ERG data from 10 or more age-matched eyes that underwent PPV for RRD. As the next best way, we chose the sound fellow eye as normal control as several previous clinical studies have done [30,31]. Fifth, ERG data recorded with conventional contact lens electrode were not available. It would be of interest to compare the ERG responses between skin electrode and corneal contact electrode. Sixth, full-field ERGs were not correlated with the visual acuity. Therefore, a study on the relationship between the ERGs and visual field may be clinically relevant to determine if the ERGs before the SO removal can predict the visual fields after the SO removal.

In conclusion, the removal of a SO tamponade increases the amplitudes of the ERGs after a period of retinal reattachment and photoreceptor recovery. These changes in the ERGs may be attributed to the non-conductive effects of SO and posterior synechia of the iris. ERG recordings with skin electrodes will allow a functional evaluation of SO filled eye. The functional evaluation on the operated eye with RETeval system in the daily practice can provide new insights on the evaluation on the surgery and several vitreoretinal pathologies and understandings of clinical electrophysiology.

## Supporting information

S1 Dataset Raw ERG wave from each case. (PDF)

S2 Dataset Values to calculate mean, standard deviation, median, and 25^th^ and 75th percentiles, and to build Fig 2 and Fig 3. (XLSX)
