# Correction: Electroretinograms recorded with skin electrodes in silicone oil-filled eyes

**DOI:** 10.1371/journal.pone.0242757

**Published:** 2020-11-17

**Authors:** Kimitake Ozaki, Yuji Yoshikawa, Sho Ishikawa, Takeshi Katsumoto, Masayuki Shibuya, Takuhei Shoji, Hiromi Kondo, Soiti Matsumoto, Kei Shinoda

In the Subjects and methods section, there is an error in the first sentence of the second paragraph. The correct sentence is: Eleven eyes of 11 patients with complex vitreoretinal disorders were studied. There were 5 men and 6 women who had undergone pars plana vitrectomy (PPV) with purified SO as a tamponade (SILIKON1000, Alcon Japan Ltd, Tokyo, Japan).

In the right panel of Fig 3 the approximate straight line of the a-wave is orange, but it should be blue. There is also information missing from the Fig 3 caption. Please see the complete, correct Fig 3 and Fig 3 caption here.

**Fig 3:**
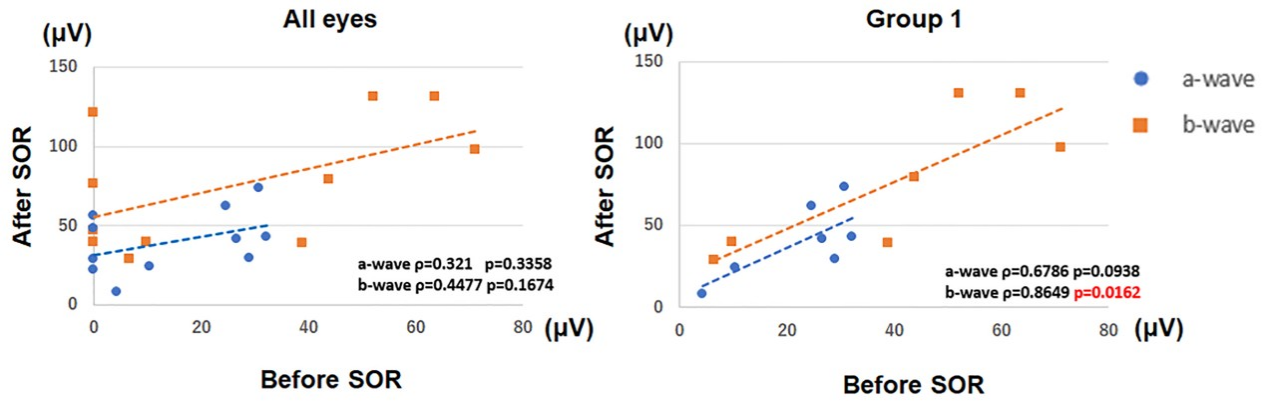
Amplitudes of the a- and b-waves before and after silicone oil (SO) removal. There was a significant positive correlation between the b-wave amplitudes before and after SO removal for the 7 eyes in Group 1 (ρ = 0.8649, P = 0.0162). SOR, silicone oil removal; blue circle, a-wave; orange square, b-wave.
