# Supplementary material for: Electroretinograms recorded with skin electrodes in silicone oil-filled eyes
Source: PLoS One. 2019 May 31;14(5):e0216823. doi: 10.1371/journal.pone.0216823 (PMC6544342; doi:10.1371/journal.pone.0216823)

CASE 1

Pre SOR

EXAMINATION **#4**

Flash: 3.0 cd·s/m², Chromaticity(0.33, 0.33), 0.1 Hz    Background light: 0.0 cd/m²

Right eye

| a wave ms | μV | b wabe ms | μV |
|---|---|---|---|
| 19.0 | -4.4 | 49.5 | 6.6 |

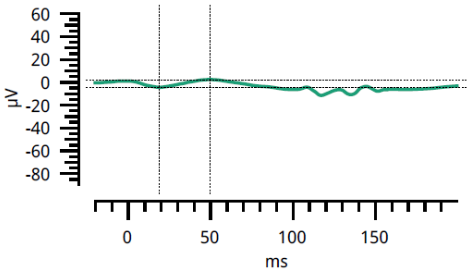


Left eye

| a wave ms | μV | b wave ms | μV |
|---|---|---|---|
| 14.0 | -60.0 | 51.4 | 81.3 |

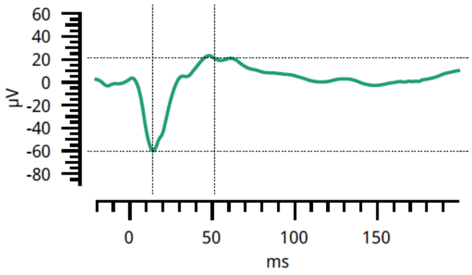


Post SOR

Examination **#4**

Flash: 3.0 cd·s/m², Chromaticity (0.33, 0.33), 0.1 Hz    Background light: 0.0 cd/m²

Right eye

| a wave ms | μV | b wave ms | μV |
|---|---|---|---|
| 20.2 | -8.1 | 87.6 | 28.7 |

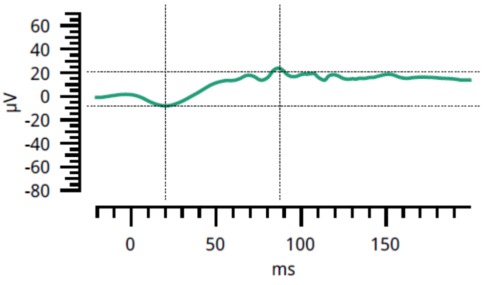


Left eye

| a wave ms | μV | b wave ms | μV |
|---|---|---|---|
| 14.2 | -36.7 | 59.3 | 60.4 |

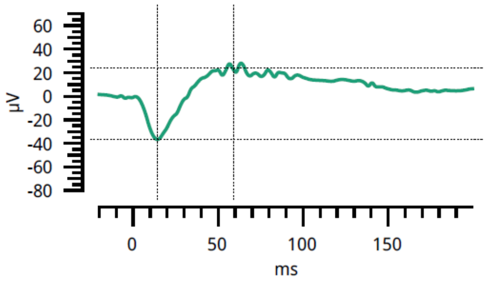

CASE　2

Pre SOR

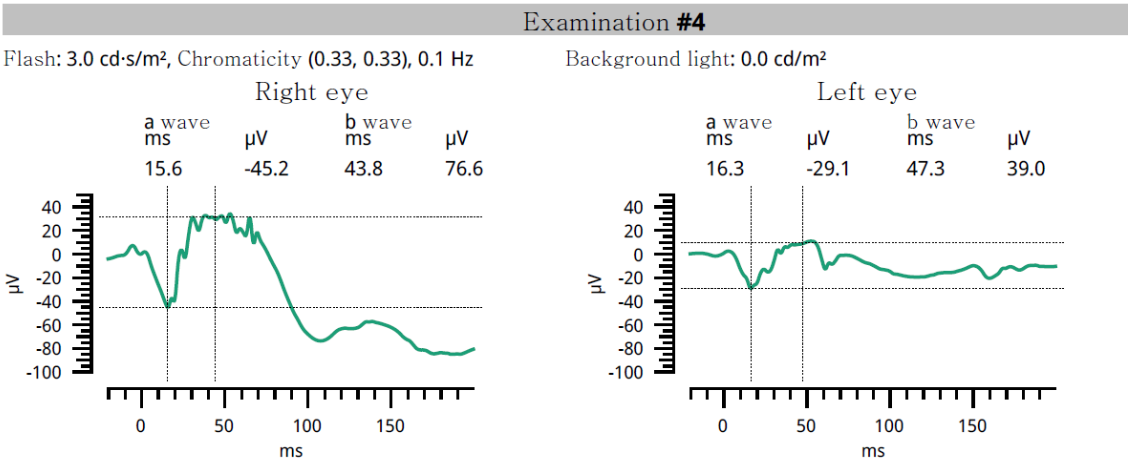


Post SOR

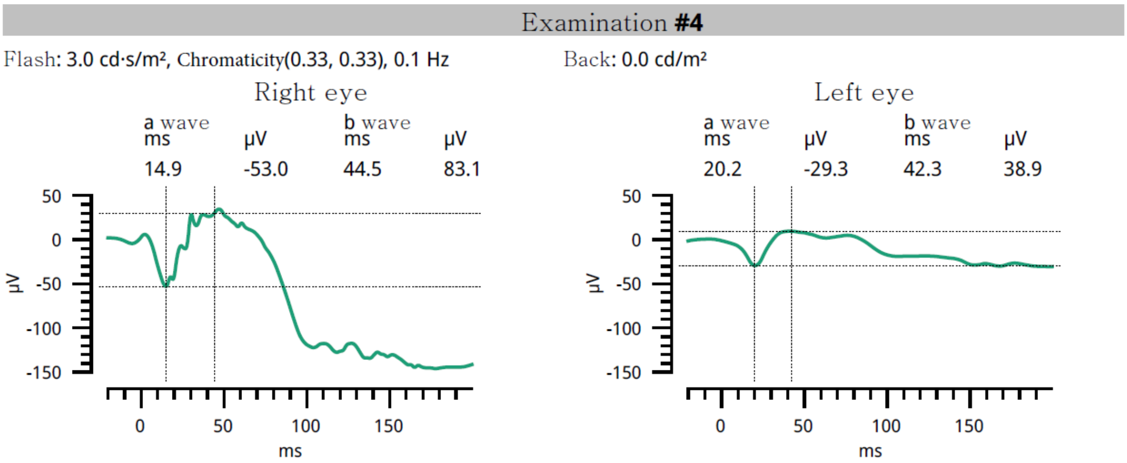

CASE　3

Pre　SOR

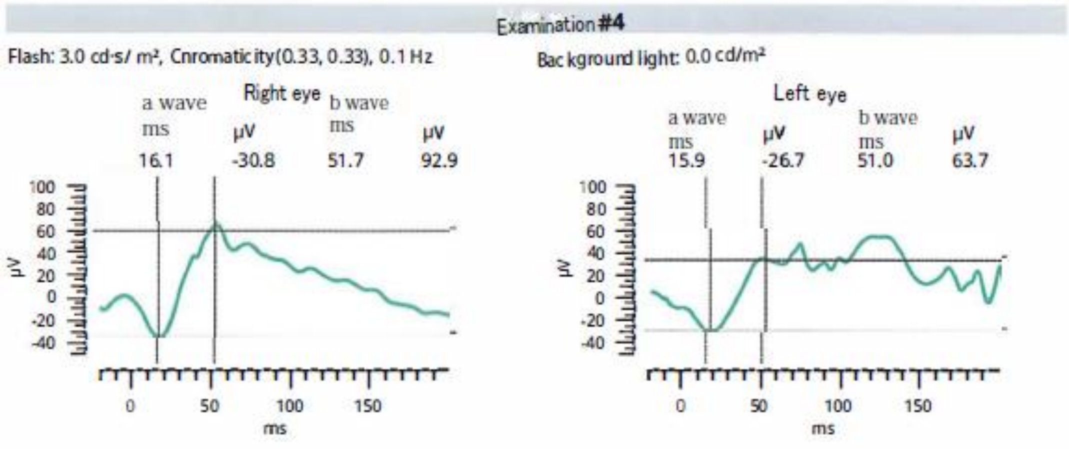


Post　SOR

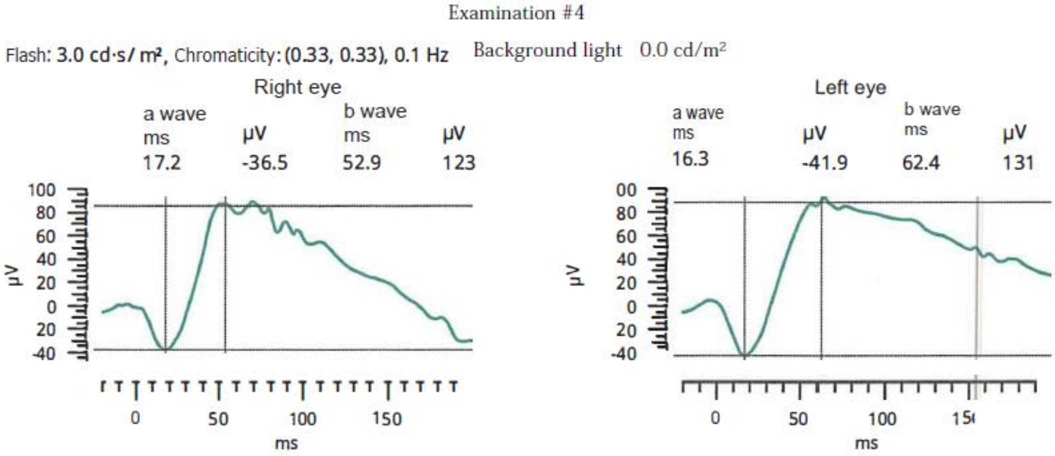

CASE　4

Pre　SOR

**Examination #4**

Flash: 3.0 cd·s/m², Chromaticity (0.33, 0.33), 0.1 Hz　　Background light: 0.0 cd/m²

| | a wave ms | µV | b wave ms | µV |
|---|---|---|---|---|
| Right eye | 20.9 | -32.2 | 54.8 | 71.3 |
| Left eye | 16.2 | -40.0 | 50.1 | 76.1 |

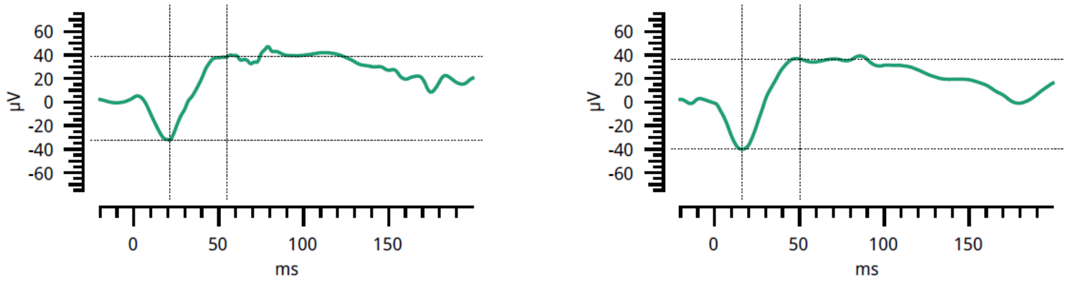


Post　SOR

**Examination #4**

Flash: 3.0 cd·s/m², Chromaticity (0.33, 0.33), 0.1 Hz　　Background light: 0.0 cd/m²

| | a wave ms | µV | b wave ms | µV |
|---|---|---|---|---|
| Right eye | 21.5 | -43.3 | 55.0 | 97.6 |
| Left eye | 17.1 | -23.6 | 70.5 | 59.9 |

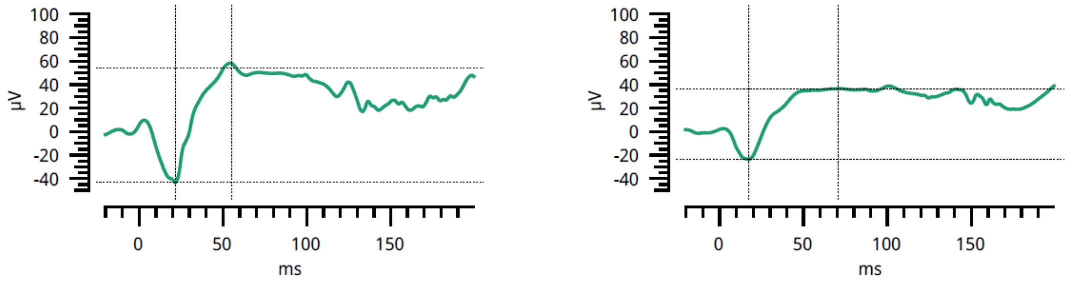

CASE　5

Pre　SOR

**Examination #1**

Flash 3.0 cd·s/m², Chromaticity (0.33, 0.33), 0.1 Hz　　Background light: 0.0 cd/m²

Right eye

| a wave ms | µV | b wave ms | µV |
|---|---|---|---|
| 36.1 | -8.2 | 45.4 | 3.6 |

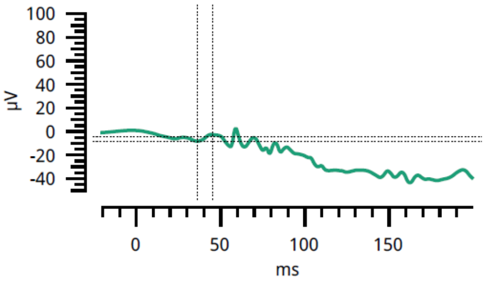


Left eye

| a wave ms | µV | b wave ms | µV |
|---|---|---|---|
| 14.0 | -47.5 | 51.7 | 100 |

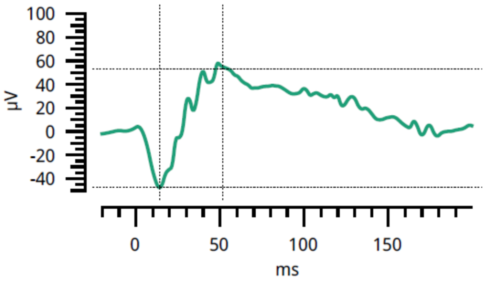


Post　SOR

**Examination #4**

Flash: 3.0 cd·s/m², Chromaticity (0.33, 0.33), 0.1 Hz　　Background light: 0.0 cd/m²

Right eye

| a wave ms | µV | b wave ms | µV |
|---|---|---|---|
| 22.2 | -56.3 | 56.5 | 121 |

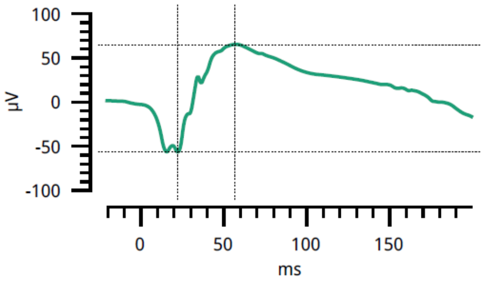


Left eye

| a wave ms | µV | b wave ms | µV |
|---|---|---|---|
| 14.6 | -58.8 | 50.8 | 125 |

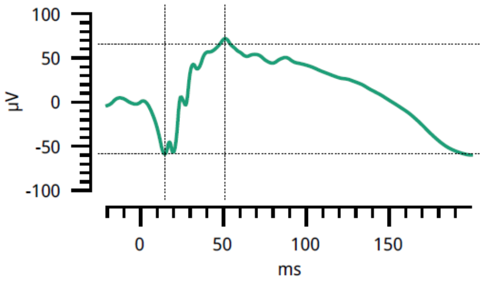

CASE　6

Pre　SOR

**Examination #1**

Flash: 3.0 cd·s/m², Chromaticity (0.33, 0.33), 0.1 Hz　　Background light: 0.0 cd/m²

Right eye

| a wave ms | µV | b wave ms | µV |
|---|---|---|---|
| 20.3 | -4.1 | Unrecordable | Unrecordable |

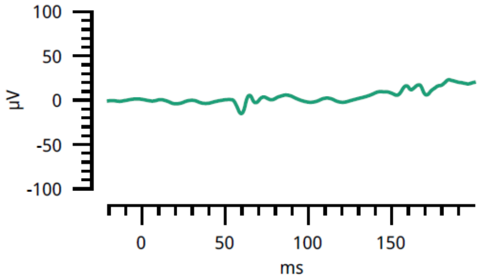


Left eye

| a wave ms | µV | b wave ms | µV |
|---|---|---|---|
| 16.5 | -52.6 | 50.7 | 111 |

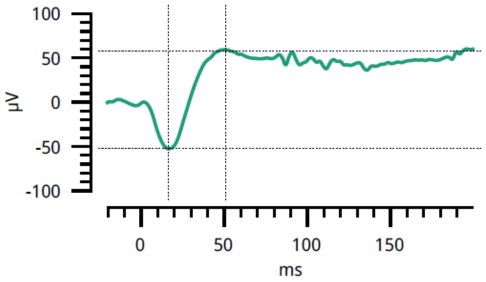


Post　SOR

Examination **#4**

Flash: 3.0 cd·s/m², Chromaticity (0.33, 0.33), 0.1 Hz　　background light: 0.0 cd/m²

Right eye

| a wave ms | µV | b wave ms | µV |
|---|---|---|---|
| 17.4 | -48.0 | 46.4 | 76.5 |

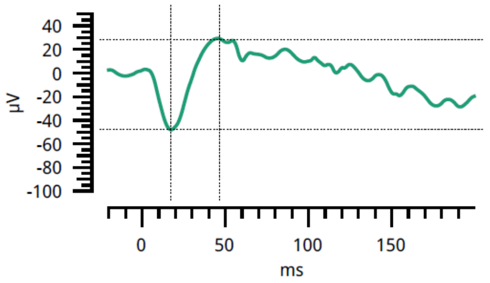


Left eye

| a wave ms | µV | b wave ms | µV |
|---|---|---|---|
| 15.8 | -65.0 | 53.0 | 111 |

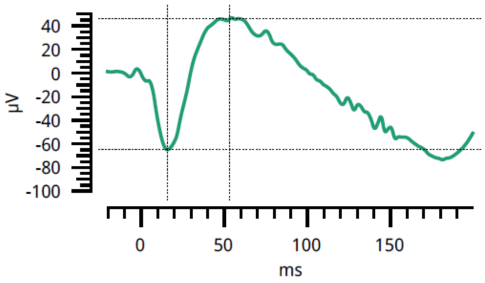

CASE　7

Pre　SOR

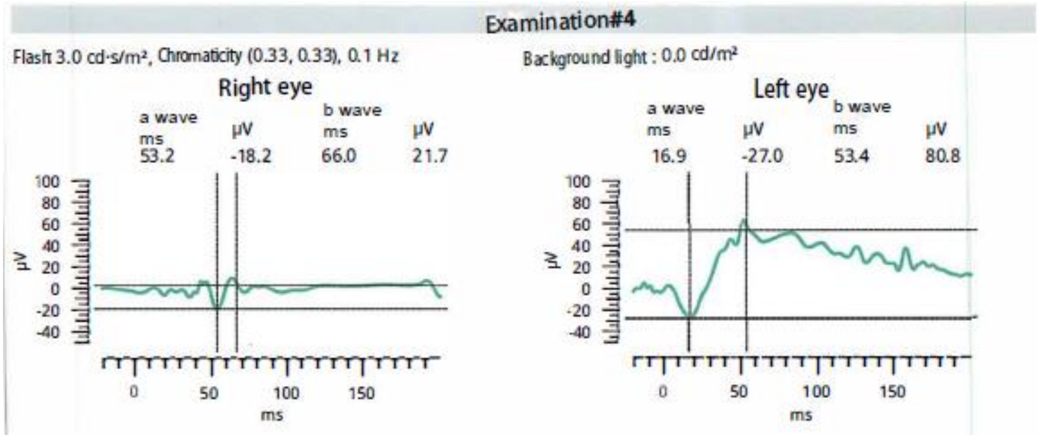


Post　SOR

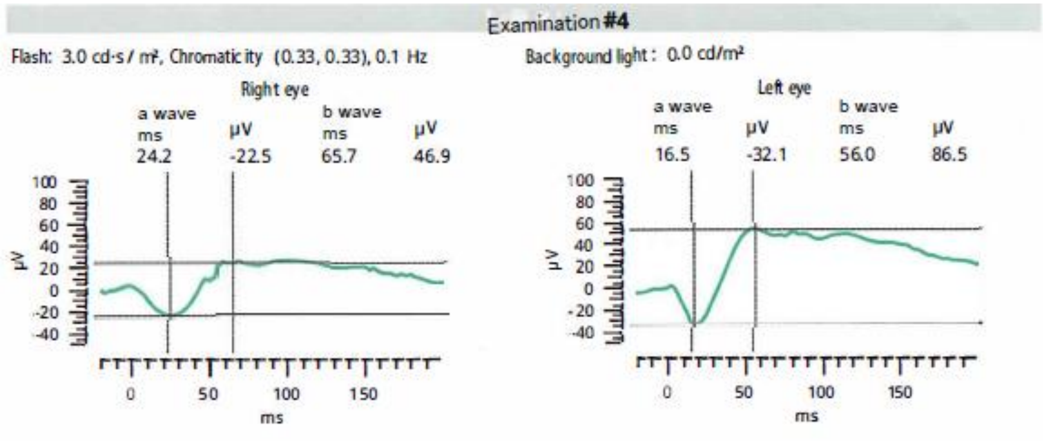

CASE　8

Pre　SOR

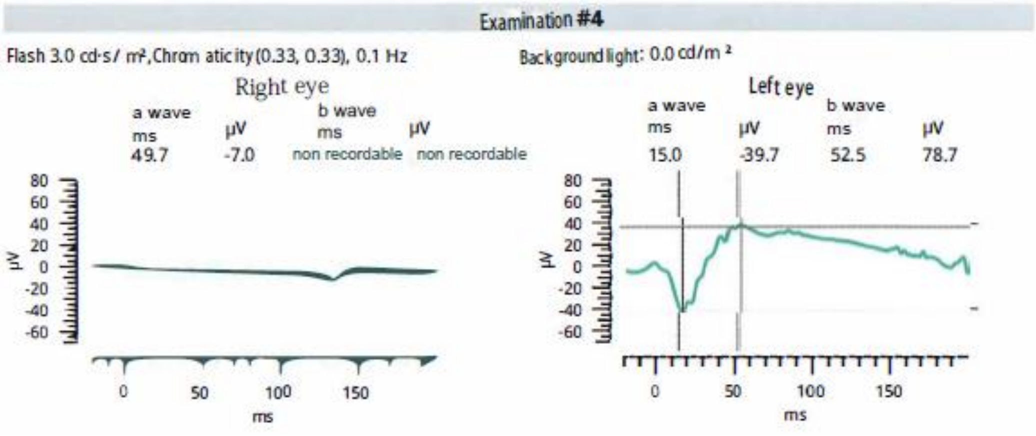


Post SOR

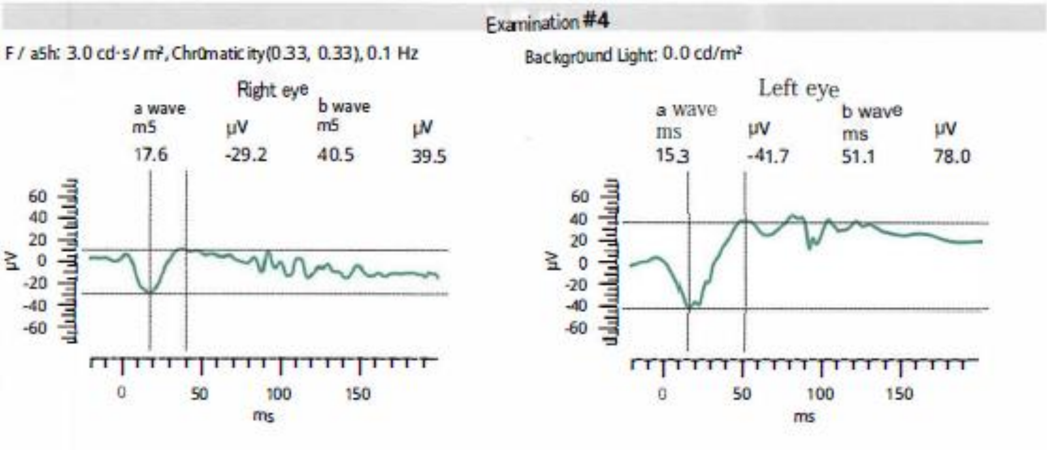

CASE　9

Pre　SOR

Examination **#4**

Flash: 3.0 cd·s/m², Chromaticity(0.33, 0.33), 0.1 Hz　　Background light: 0.0 cd/m²

Right eye

| a wave ms | µV | b wave ms | µV |
|---|---|---|---|
| 17.4 | -10.4 | 42.6 | 9.9 |

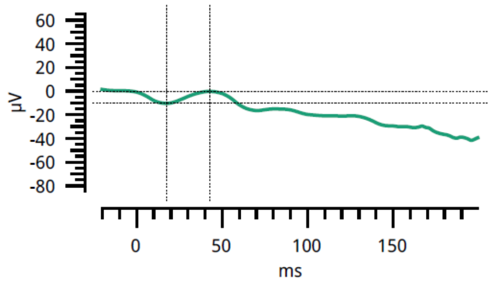


Left eye

| a wave ms | µV | b wave ms | µV |
|---|---|---|---|
| 14.5 | -25.0 | 46.9 | 45.5 |

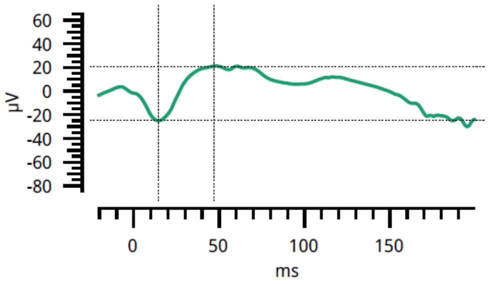


Post　SOR

**Examination#4**

Flash: 3.0 cd·s/m², Chromaticity (0.33, 0.33), 0.1 Hz　　Background light: 0.0 cd/m²

Right eye

| a wave ms | µV | b wave ms | µV |
|---|---|---|---|
| 16.1 | -24.6 | 50.6 | 39.9 |

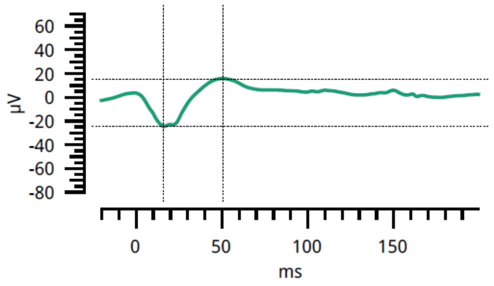


Left eye

| a wave ms | µV | b wave ms | µV |
|---|---|---|---|
| 14.4 | -40.7 | 53.3 | 74.3 |

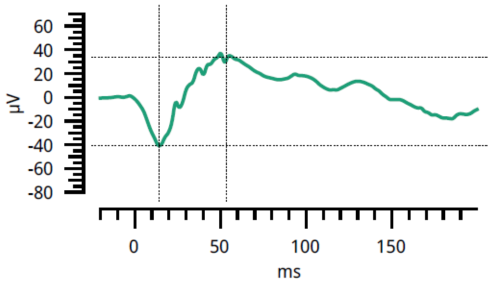

CASE　１０

Pre　SOR

**Examination #4**

Flash: 3.0 cd·s/m², Chromaticity (0.33, 0.33), 0.1 Hz　　Background: 0.0 cd/m²

Right eye

| a wave ms | μV | b wave ms | μV |
|---|---|---|---|
| 16.4 | -51.1 | 60.1 | 84.6 |

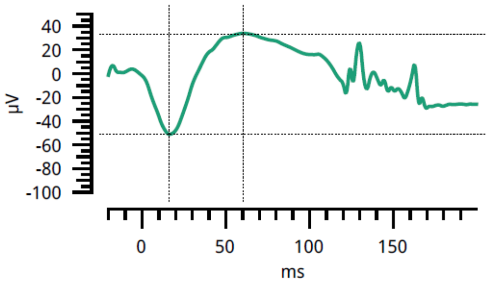


Left eye

| a wave ms | μV | b wave ms | μV |
|---|---|---|---|
| 20.6 | -24.7 | 59.8 | 43.9 |

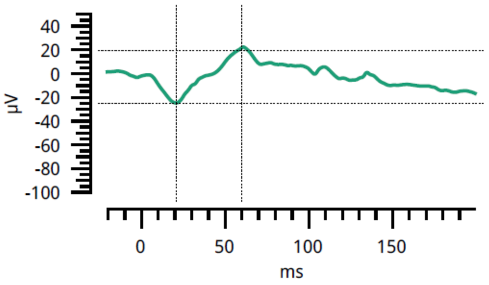


Post　SOR

Examination **#4**

Flash: 3.0 cd·s/m², Chromaticity (0.33, 0.33), 0.1 Hz　　Background light: 0.0 cd/m²

Right eye

| a wave ms | μV | b wave ms | μV |
|---|---|---|---|
| 16.0 | -53.4 | 53.5 | 90.7 |

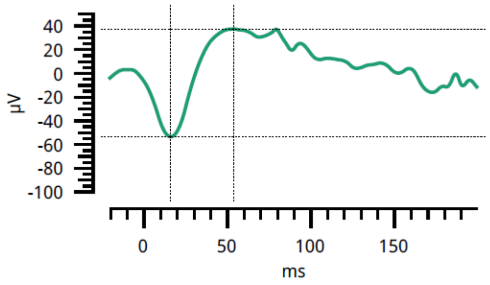


Left eye

| a wave ms | μV | b wave ms | μV |
|---|---|---|---|
| 16.9 | -62.2 | 64.4 | 79.3 |

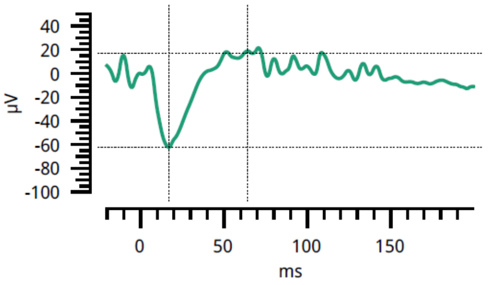

CASE　１１

Pre　SOR

## Examination #4

Flash: 3.0 cd·s/m², Chromaticity (0.33, 0.33), 0.1 Hz　　Background light: 0.0 cd/m²

### Right eye

| a wave ms | µV | b wave ms | µV |
|---|---|---|---|
| 21.3 | -44.2 | 52.5 | 87.2 |

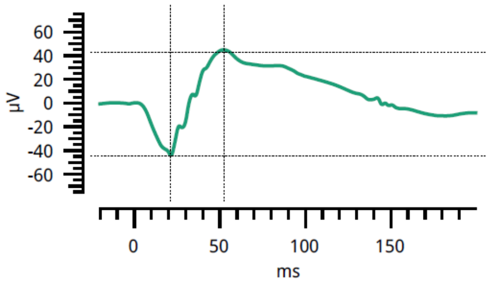


### Left eye

| a wave ms | µV | b wave ms | µV |
|---|---|---|---|
| 18.4 | -30.8 | 53.6 | 52.2 |

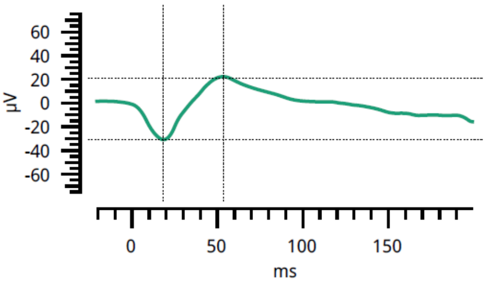


Post　SOR

## Examination #4

Flash: 3.0 cd·s/m², Chro (0.33, 0.33), 0.1 Hz　　Background light: 0.0 cd/m²

### Right eye

| a wave ms | µV | b wave ms | µV |
|---|---|---|---|
| 21.6 | -49.5 | 53.6 | 87.4 |

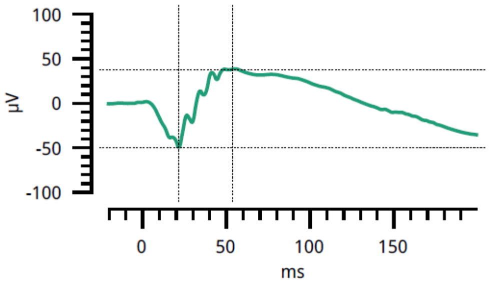


### Left eye

| a wave ms | µV | b wave ms | µV |
|---|---|---|---|
| 21.4 | -73.6 | 59.6 | 131 |

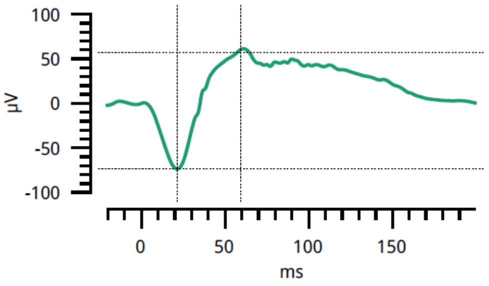

Supplement: S1 Dataset — (PDF) [file pone.0216823.s001.pdf]
